# The Proteolytic Cleavage of Therapeutic Monoclonal Antibody Hinge Region: More Than a Matter of Subclass

**DOI:** 10.3389/fimmu.2020.00168

**Published:** 2020-02-11

**Authors:** Quentin Deveuve, Laurie Lajoie, Benjamin Barrault, Gilles Thibault

**Affiliations:** ^1^EA7501 Groupe Innovation et Ciblage Cellulaire, Equipe Fc Récepteurs, Anticorps et MicroEnvironnement, Université de Tours, Tours, France; ^2^Laboratoire d'Immunologie, CHRU de Tours, Tours, France

**Keywords:** therapeutic monoclonal antibodies, hinge region, proteolytic cleavage, MMP12, ideS, immunoglobulin G subclass, C1q, FcγRIIIA

## Abstract

The hinge region of immunoglobulin G (IgG) is involved in C1q and FcγRIIIA-expressing natural killer (NK) cell recruitment. Both heavy chains (HCs) of the hinge region can be cleaved sequentially by several proteases of the tumor/inflammatory/infectious microenvironment, including matrix metalloproteinase 12 (MMP12), or immunoglobulin-degrading enzyme from *Streptococcus pyogenes* (IdeS), impairing Fc-mediated functions. The cleavage of therapeutic monoclonal antibodies (TmAbs), which are based on a human IgG1, IgG2 or IgG4 structure, has been poorly investigated, although it may represent an escape mechanism to these treatments. Therefore, we used non-reducing SDS-PAGE to compare the cleavage kinetics of five IgG1 TmAbs (trastuzumab, rituximab, cetuximab, infliximab, ipilimumab), one IgG2 TmAb (panitumumab), and two IgG4 TmAbs (nivolumab and pembrolizumab) by MMP12 and IdeS, which were found to cleave the first and second HCs with different kinetics. Panitumumab was more protease-resistant than IgG1 and IgG4 TmAbs. The latter were usually more protease-sensitive, whereas IgG1 TmAbs were usually cleaved with intermediate kinetics. However, we observed intra-subclass variability among IgG4 and IgG1 TmAbs. Nivolumab and pembrolizumab were cleaved similarly by MMP12, whereas pembrolizumab was more IdeS-resistant. Ipilimumab was more IdeS-sensitive and MMP12-resistant than the other IgG1 TmAbs, regardless of G1m allotype. In addition the Fc fragment of IgG1 TmAbs were highly resistant to cleavage by MMP12, whereas their cleavage kinetic by IdeS was very similar to that observed with the intact forms (excluding ipilimumab). Importantly, the cleavage kinetic of ipilimumab Fc fragment by IdeS was superimposable to that of trastuzumab, cetuximab and infliximab Fc fragment, showing that the variability observed for intact ipilimumab is unrelated to its Fc portion. We propose that the variability in the cleavage sensitivity/resistance balance among TmAbs of IgG1 and IgG4 subclasses results partially, from TmAb characteristics related to and/or located in the Fab region. Finally, with ELISA and flow cytometry, we observed that a single cleavage of IgG1 TmAbs greatly decreased their affinity for FcγRIIIA and C1q and their ability to induce FcγRIIIA-dependent functional responses of NK cells. Overall, our results indicate that the cleavage of the hinge region should be considered with TmAbs treatment and in the development of new molecules.

## Introduction

Therapeutic monoclonal antibodies (TmAbs), which are extensively used for treatment in cancer or chronic inflammatory diseases, are based on a human IgG1, IgG2, or IgG4 structure. The choice among the three subclasses during the design and development of an mAb is mainly directed by its expected mechanism of action. The IgG1 format efficiently binds C1q and FcγRs and triggers complement-dependent cytotoxicity or antibody-dependent cell-mediated cytotoxicity (ADCC) (1, 2). Therefore, this subclass is used to develop cytolytic mAbs. Conversely, the IgG2 or IgG4 format, which weakly binds C1q and FcγR, is usually favored when developing neutralizing/antagonist TmAbs. The binding sites of C1q and FcγR on human IgG are relatively close and are partially located in the lower hinge region (defined by the sequence ^233^PAPELLGGP^241^ in IgG1) (1–3).

Previous studies have shown that in addition to the papain and pepsin sensitivity of the hinge region (4, 5), the lower hinge region of IgG1 is cleaved by tumor-, inflammatory-, and/or infectious-associated proteases such as matrix metalloproteinases (MMPs) or immunoglobulin-degrading enzyme from *Streptococcus pyogenes* (IdeS) (6–11). A first cleavage of one heavy chain (HC) generates single-cleaved IgG (sc-IgG), which retains its whole structure via CH3-CH3 and glycan weak interactions (9). Nevertheless, the cleavage sites are located within or proximal to the binding sites for C1q and FcγR, thus reducing their binding and ultimately Fc-mediated functional responses (1, 2, 6). The cleavage of the second HC is usually slower and generates F(ab')_2_, which obviously loses the Fc-dependent interactions. The cleavage of trastuzumab or pertuzumab (anti-HER2, used in breast cancer therapy) impairs ADCC and has been associated with a weakened therapeutic effect in a mouse xenograft tumor model (12, 13). Furthermore, high levels of cleaved IgG were detected in serum from individuals with inflammatory bowel diseases, who did not respond to anti-TNFα TmAbs (10). Therefore, the proteolytic cleavage could be an immune evasion mechanism in IgG1 TmAbs treatment.

By contrast, IgG2s are almost completely MMP-resistant and only partially cleaved by IdeS. This resistance has been linked to the sequence of their lower hinge region. Indeed, replacing the ^234^PELLGG^240^ (EU numbering) of an IgG1 mAb hinge region with the ^231^PPVA-G^236^ sequence of an IgG2 resulted in complete resistance to the cleavage by MMPs (14). Finally, the cleavage of IgG4 has been poorly documented, although Ryan et al. suggested that IgG1 and IgG4 mAbs were similarly cleaved by MMP3 and IdeS (6). Besides the amino acid composition of the lower hinge region related to the subclasses, the numerous available TmAbs feature other structural and/or conformational variations. Some are natural, such as the four IgG1 allotypes resulting from the combination of genetic markers (Gms) (K214/R214 and D356-L358/E356-M358 defining the G1m17/G1m3 and G1m1/G1m-1 allotypes, respectively) or Fc glycosylation. Other variations are related to bioengineering such as the type of humanization (chimeric, humanized, or full human) or the S228P mutation introduced to stabilize the core hinge region of IgG4 (15). The effect of this subclass-independent structural heterogeneity of engineered TmAbs on protease sensitivity is unknown.

In this context, we aimed to compare the cleavage of a panel of eight widely used TmAbs (three chimeric, two humanized, and three full-human) regrouping five IgG1s that include the four allotypes, one IgG2 TmAb, and two IgG4 TmAbs. We chose IdeS as a model protease (12–14, 16) and MMP12, secreted by macrophages in the tumor microenvironment or in chronic inflammatory diseases, as a more relevant pathophysiological protease. We used SDS-PAGE to kinetically analyze the proteolytic fragments for each TmAbs. Moreover, we analyzed the effect of the cleavage on the binding of C1q and membrane FcγRIIIA as well as its functional consequences by ELISA or multicolor flow cytometry.

## Materials and Methods

### Monoclonal Antibodies

TmAbs obtained from the CHRU de Tours included five IgG1 molecules: cetuximab (anti-EGFR), infliximab (anti-TNFα), ipilimumab (anti-CTLA4), rituximab (anti-CD20), trastuzumab (anti-HER2), one IgG2 (panitumumab [anti-EGFR]), and two IgG4 molecules (nivolumab and pembrolizumab [both anti-PD1]). MAbs including unconjugated anti-CD16 (clone 3G8) and FITC-conjugated anti-CD16 (clone 3G8) and PE-conjugated anti-IFNγ (clone 45.15) were from Beckman Coulter and PE-Cy5-conjugated CD107a (clone H4A3) was from BD Biosciences. Horseradish peroxidase-conjugated anti-C1q was from Abcam. Allotypes of rituximab were produced by Evitria (Schlieren, Switzerland) were constructed following the same strategy as described by Ternant et al. (17).

### Enzymes and Proteolytic Cleavage

Recombinant IdeS and IgdE were from Genovis. The human recombinant MMP12 catalytic domain was from Sinobiological Inc. The proteolytic cleavage of 50 μg TmAbs (1 mg/mL) involved 0.05 U IdeS/μg TmAbs or 10 μg/mL MMP12 in phosphate buffered saline (PBS, pH 7.4) and tris buffered saline (pH 7.5), respectively. MMP12 activity required the addition of 10 mM CaCl_2_. At the indicated times, a sample of the reaction was stopped by 10 mM iodoacetamide (Sigma Aldrich) for IdeS or 1 mM EDTA for MMP12. To generate single-cleaved TmAbs (sc-TmAbs) for binding and functional experiments, TmAbs were incubated at 3 mg/mL with 0.1 U IdeS/μg TmAbs in PBS for 6 min. All reactions were performed at 37°C.

In order to produce Fc fragments, 50 μg TmAbs were incubated with 1 U IgdE/μg TmAbs either in PBS (pH 7.4) or TBS (pH 7.5) at 37°C for 48 h.

### SDS-PAGE

Proteolytic fragments were analyzed by SDS-PAGE in NuPAGE MES SDS running buffer (ThermoScientific) on 6% (or 10% for the cleavage of the Fc domain) homemade gels under non-reducing conditions. Samples were heated at 95°C for 10 min in Laemmli buffer before electrophoresis. SeeBlue Plus2 Pre-stained Protein Standard (Invitrogen) was used as a weight molecular marker. Proteins were stained with Coomassie Blue and images were acquired on a Fusion Fx (Vilber Lourmat). Relative percentages of each fragment were obtained by densitometry using ImageJ (1.50i) software.

### Binding Property of TmAbs to FcγRIIIA

CD16-transduced NK92 cells (2 × 10^4^) were incubated with FITC-conjugated anti-CD16 3G8 (dilution 1:100) and increasing concentrations of TmAbs or sc-TmAbs (30 min at 4°C) and were analyzed by flow cytometry as described (18).

### Functional Responses of CD16-Transduced NK92 Cells

IgG2 mouse mAb, mouse anti-CD16 mAb, or (sc-)TmAbs (5 μg/mL) was used to sensitize Nunc Maxisorp 96-well culture plates (ThermoScientific) overnight at 4°C. Then, 100 μL CD16-transduced NK92 cells (2 × 10^4^) were plated on non-sensitized or sensitized plates and incubated at 37°C in 5% CO_2_ humidified air for 4 h with anti-CD107a mAb (dilution 1:20) and 0.1 μg/mL BD GolgiPlug containing Brefeldin A (BD Biosciences). Cells were fixed and permeabilized by using the BD Cytofix/cytoperm Plus kit (BD Biosciences) and stained with anti-IFNγ mAbs (dilution 1:10) for 30 min at 4°C. Data were acquired with a Gallios flow cytometer (Beckman Coulter) and were analyzed by using Kaluza v1.3.

### C1q ELISA

Nunc Maxisorp 96-well culture plates were coated with saturating concentrations of (sc-)TmAbs (10 μg/mL) in 0.05 mol/L bicarbonate buffer (pH 9.5) overnight at 4°C. Wells were washed three times with PBS containing 0.05% Tween-20, then blocked with PBS-0.05% Tween-1% BSA for 1 h at 37°C. After three washes, 0.01–30 μg/mL C1q was added. After incubation for 2 h at 37°C, wells were washed three times with PBS-0.05% Tween-20 and incubated with horseradish peroxidase-conjugated anti-C1q (dilution 1:200) for 1 h at 37°C. 3,3′,5,5′-Tetramethylbenzidine (Thermo Scientific) was used as a substrate. Absorbance was measured at 405 nm on a Mithras LB940 (Berthold Technologies).

## Results

### Comparison of MMP12- and IdeS-Mediated Cleavage of TmAbs

**Figure 1:**
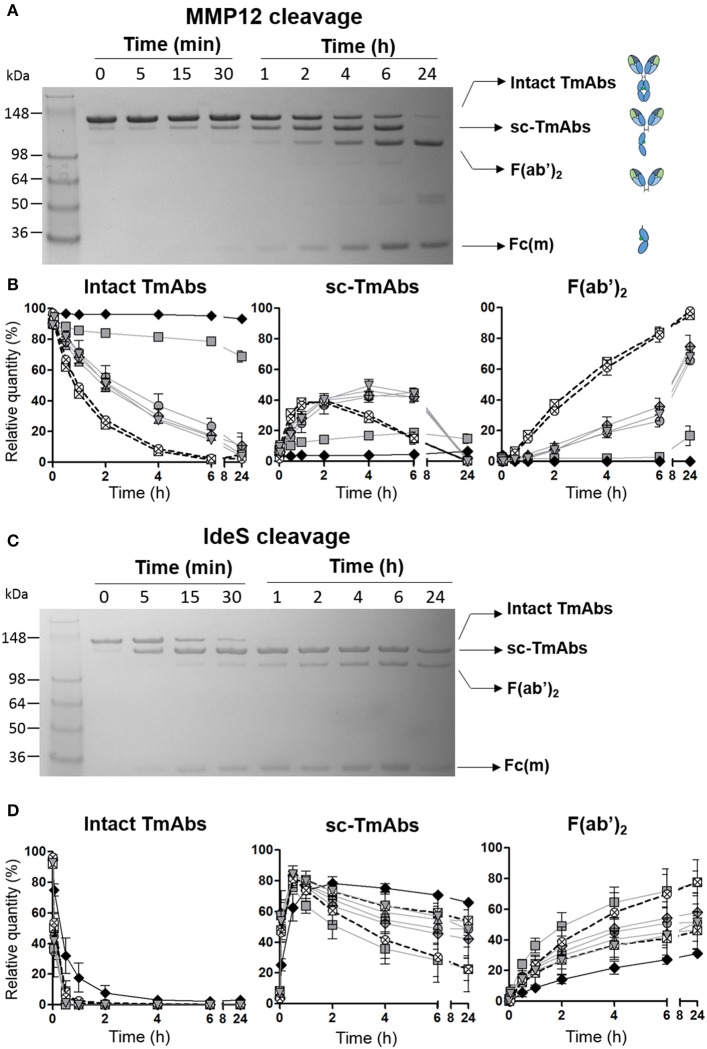
Cleavage of TmAbs by MMP12 or IdeS. Non-reducing SDS-PAGE for rituximab without or with MMP12 **(A)** or IdeS **(C)** incubation (representative example). **(B,D)** show the relative quantity of cleavage product: intact TmAbs (left), single-cleaved forms (middle), and F(ab')_2_ (right) at different times after incubation with MMP12 **(B)** or IdeS **(D)**. The eight TmAbs (IgG1 in gray line, IgG2 black line, and IgG4 dotted line) are indicated: trastuzumab (circle), cetuximab (triangle), infliximab (crossed diamond), ipilimumab (square), rituximab (inverted triangle), panitumumab (diamond), nivolumab (crossed circle), and pembrolizumab (crossed square). Each band was quantified by densitometry with ImageJ. Data are mean (SD) (*n* = 3).

To kinetically compare the proteolytic cleavage of TmAbs, we first incubated five IgG1 TmAbs (trastuzumab, rituximab, cetuximab, infliximab, and ipilimumab), one IgG2 TmAb (panitumumab), and two IgG4 TmAbs (nivolumab and pembrolizumab) with MMP12 for various times. The generated fragments were analyzed by non-reducing SDS-PAGE. An example of the cleavage (rituximab) is shown in Figure 1A. The ≈148 kDa band (intact rituximab) started to decrease after 5–15 min and almost completely disappeared after 6 h. After 15 min, two bands corresponding to the cleavage of one HC were detected: one ≈125 kDa (sc-TmAbs) and one ≈30–35 kDa [hemi-Fc (Fc(m)) released in denaturing conditions]. The sc-TmAb band increased up to 4–6 h and disappeared at 24 h, while the Fc(m) band increased over time. A fourth band of ≈98 kDa (F(ab')_2_, corresponding to the cleavage of the second HC, was observed after 30 min. It increased with time and was the major band detected after 24 h. Finally, two additional slight bands of ≈50–55 kDa were observed after 4 h when IgG1 TmAbs were tested. The bands corresponding to the intact TmAbs, sc-TmAbs and F(ab')_2_ were quantified (Figure 1B).

The IgG2 mAb panitumumab was highly resistant to cleavage by MMP12: the intact form represented >90% after 24 h (Figure 1B left). By contrast, intact forms of the two IgG4 TmAbs pembrolizumab and nivolumab decreased rapidly and disappeared after 6 h. Sc-TmAbs increased rapidly (up to ≈40% at 1–2 h), then decreased and became undetectable after 24 h (Figure 1B middle). The proportion of F(ab')_2_ increased constantly to represent >80 and >90% after 6 and 24 h, respectively (Figure 1B, right). Finally, among IgG1 TmAbs, trastuzumab, rituximab, cetuximab, and infliximab showed an intermediate cleavage kinetics: about 20% of the intact form still observed after 6 h of incubation, with the maximum of sc-TmAbs (up to ≈40%) observed at 4–6 h, followed by almost complete disappearance. The proportion of F(ab')_2_ increased constantly to represent ≈30 and >80% after 6 and 24 h, respectively (Figure 1B, middle and right). Unexpectedly, the cleavage of ipilimumab, the fifth IgG1 TmAb, was markedly slower and close to that of panitumumab: the intact form was still ≈70% after 24 h and the sc-TmAb and F(ab')_2_ forms did not exceed 20%, whatever the time.

We then studied cleavage by IdeS. As shown in Figure 1C for rituximab and Figure 1D for all TmAbs, the cleavage by IdeS was actually faster than that by MMP12 (Figure 1D, left). IdeS cleaved the first HC very rapidly: almost all intact forms were converted in sc-TmAbs (Figure 1D, middle) when F(ab')_2_ became detectable (Figure 1D, right). However, the latter form did not exceed 75% after 24 h, whatever the TmAb. Finally, we did not detect any additional bands as observed in MMP12 experiments.

The proteolytic profile of the eight TmAbs with IdeS according to their subclass differed from that observed with MMP12. First, the cleavage of the first HC (leading to sc-TmAb) was similar whatever the TmAb studied, except for the panitumumab: maximal sc-panitumumab proportion was observed after ≈2 h vs. 30 min for the other TmAbs (Figure 1D, middle). Therefore, the differences between the TmAbs mainly affected the cleavage of the second HC, as observed by the simultaneous decrease of sc-TmAbs and increase of F(ab')_2_ forms (Figure 1D, middle and right). The panitumumab second HC was highly resistant to this cleavage by IdeS [≈30% of F(ab')_2_ at 24 h]. Secondly, the two IgG4 TmAbs did not behave similarly. The cleavage of the second HC of pembrolizumab was slower than that of nivolumab: F(ab')_2_ increased from ≈20 to 45% between 1 and 24 h and from ≈25 to 75% between 1 and 24 h. Finally, the cleavage of the second HC of trastuzumab, rituximab, cetuximab and infliximab was similar [≈40–50% of sc-TmAbs and F(ab')_2_ at 24 h] and intermediate between that of IdeS-resistant panitumumab and IdeS-sensitive nivolumab. The profile of ipilimumab was once again different from that of the other IgG1-TmAbs and was close to that of nivolumab. Indeed, after 30 min, sc-ipilimumab decreased, leaving only ≈20% of this form and ≈80% of F(ab')_2_ after 24 h (Figure 1D).

**Figure 2:**
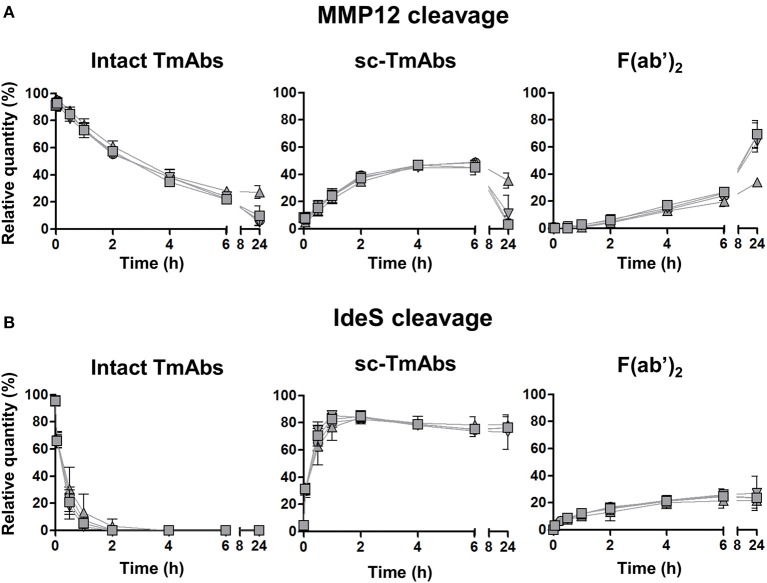
Cleavage of rituximab allotypes by MMP12 or IdeS. The relative quantity of intact TmAbs (left panel), single-cleaved forms (middle panel) and F(ab')_2_ (right panel) of the four allotypes based on the rituximab format G1m3;-1 (inverted triangle), G1m3;1 (square), G1m17;-1 (circle) or G1m17;1 (triangle) after incubation with MMP12 **(A)** or IdeS **(B)**, as described in Figure 1. Data are mean (SD) (*n* = 3).

The five IgG1 TmAbs included the four possible G1m allotypes, that is, G1m3;-1 (cetuximab), G1m3;1 (ipilimumab), G1m17;-1 (infliximab and trastuzumab), and G1m17;1 (rituximab). We wondered whether the G1m allotype could be related to the variability of IgG1 TmAbs cleavage. We compared the cleavage kinetics by MMP12 (Figure 2A) and IdeS (Figure 2B) of the four allotypes constructed on the rituximab structure. Their general profiles were very similar to those of IgG1 TmAbs excluding ipilimumab. The cleavage kinetics of the four allotypes by IdeS was superimposable, as was that with MMP12 until 6 h. After 24 h, we detected 27% of the G1m17;1 intact form vs. only 5–10% of the other mAbs (Figure 2A, left panel). Accordingly, the amount of both sc-mAbs and F(ab')_2_ of G1m17;1 was reduced (Figure 2A, middle and right). The results show the limited influence of the G1m allotype on the MMP12- and IdeS-mediated cleavage of mAbs.

Overall, the ranking of MMP12- and IdeS-mediated TmAbs cleavage (IgG4 TmAbs > IgG1 TmAbs > ipilimumab > IgG2 TmAb and nivolumab=ipilimumab > IgG1 TmAbs ≥ pembrolizumab > IgG2 TmAb, respectively) clearly differed, which shows that the proteolytic profile of a TmAb on incubation with a given protease may differ from that of other TmAbs of the same subclass.

### MMP12 and IdeS-Mediated Cleavage of IgG1 TmAbs Fc Fragment

**Figure 3:**
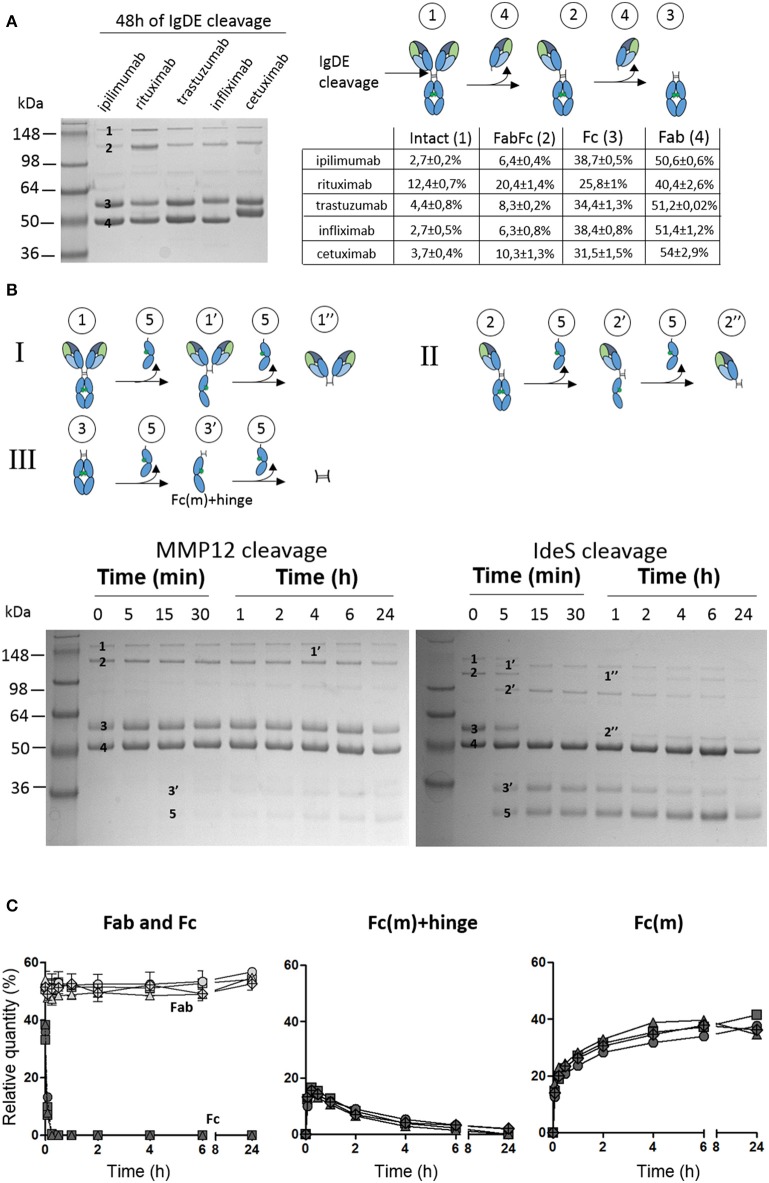
Cleavage of TmAbs Fc domain by MMP12 or IdeS. **(A)** Non-reducing SDS-PAGE for IgG1 TmAbs following IgdE cleavage. The cleavage reaction is represented on the right. The percentages of each fragment generated are also noted. **(B)** Potential cleavage reaction of IgdE-generated fragments after MMP12 or IdeS cleavage. The non-reducing SDS-PAGE for ipilimumab (representative) with or without MMP12 (left) or IdeS (right) incubation are shown. **(C)** Show the relative quantity of cleavage product: Fab (light gray) and Fc (dark gray) (left), Fc(m)+hinge (middle), and Fc(m) (right) at different times after incubation with IdeS. The generated fragments of trastuzumab (circle), cetuximab (triangle), infliximab (crossed diamond), ipilimumab (square) were quantified by densitometry with ImageJ. Data are representatives of two different experiments.

We then wondered whether the variability of IgG1 TmAbs cleavage could be related to their Fc portion. To this end, IgG1 TmAbs were firstly incubated for 48 h with IgdE, which cleaves the upper hinge region (19), followed by MMP12 and IdeS cleavage. The fragments obtained after IgdE cleavage were analyzed as described in Figures 1, 2. We observed four bands for each TmAbs: one ≈148 kDa (intact TmAbs), one ≈120 kDa corresponding to the cleavage of the first HC upper hinge region by IgdE (FabFc), one ≈55 kDa corresponding to the cleavage of the second HC upper hinge region (Fc) and finally one ≈50 kDa corresponding to the Fab (Figure 3A left). It is of note that cetuximab Fab domains are glycosylated explaining the slightly higher MW of its Fab compared to other IgG1 TmAbs. The intensity of the Fab and Fc bands was substantially higher than that of intact TmAbs or FabFc bands, whatever the TmAbs, showing that IgdE cleaved the upper hinge region efficiently in our conditions. The percentage of intact/FabFc TmAbs were very low (ranging from 8 to 14%) after cleavage of ipilimumab, trastuzumab, infliximab and cetuximab, while it reached ≈30% after cleavage of rituximab. Given this discrepancy, which may be related to the A221V mutation located very close to the upper hinge region of rituximab (20), this TmAb was not included in the following experiments.

The sequential cleavage by IdeS and MMP12 of the lower hinge region of intact and FabFc TmAbs results in the production of Fc(m) associated with several fragments >50 kDa (Figure 3B, reaction I and II). Moreover, cleavage of the first HC of a Fc fragment results in the simultaneous production of Fc(m) and Fc(m)+hinge (band ≈35 kDa), whereas cleavage of the second HC results in the disappearance of the latter and production of Fc(m) (Figure 3B, reaction III). Given that Fc(m) may results from cleavage of intact TmAbs, FabFc or Fc fragment, its monitoring is obviously insufficient to evaluate the cleavage of the Fc. However, the first and second cleavage of the Fc portion may be accurately monitored by evaluating the intensity over time of the Fc and Fc(m)+hinge bands, respectively.

Using this approach, we unexpectedly found that the intensity of the Fc band was almost unchanged over time after MMP12 incubation. Accordingly, almost no Fc(m)+hinge was observed even after 24 h of incubation, whatever the TmAb (the example of ipilimumab is shown in Figure 3B, left). This result demonstrates the inability of MMP12 to cleave the Fc fragment of the four tested IgG1 TmAbs. By contrast, the Fc decreased very rapidly and disappeared after 15 min on incubation with IdeS. This was associated with a simultaneous rapid increase of Fc(m)+hinge and Fc(m) (reflecting the cleavage of the first HC). It was followed after 30 min by the progressive decrease (and disappearance at 24 h) of the Fc(m)+hinge and increase of the Fc(m) (reflecting the cleavage of the second HC). We then compared the cleavage kinetics of the four IgG1 TmAbs (Figure 3C). The amount of Fab was unchanged over 24 h showing that the IgdE remaining in the medium did not further cleave the Fab-containing fragments (Figure 3C, left panel). Interestingly, the cleavage kinetic of the Fc by IdeS was very similar to that observed with intact IgG1 TmAbs excluding ipilimumab (shown in Figure 1D): the cleavage of the first HC was almost completed after ≈15 min [all Fc were converted in Fc(m)+hinge and Fc(m)], while the cleavage of the second HC was slower, increasing progressively from ≈ 30 min up to 24 h (reflected by the simultaneous decrease from ≈20 to 0% of Fc(m)+hinge and increase from ≈20 to 35–40% of Fc(m) (Figure 3C middle and right panel). Finally, and importantly, the cleavage kinetics of the four IgG1 TmAbs tested, including ipilimumab, were superimposable.

Overall, these results show that ipilimumab Fc fragment and that of trastuzumab, cetuximab, and infliximab were cleaved similarly by IdeS, whereas MMP12 was inefficient in our conditions.

### Effect of a Single Cleavage of TmAbs on Binding to Membrane FcγRIIIA and FcγRIIIA-Dependent Functional Responses of NK Cells

**Figure 4:**
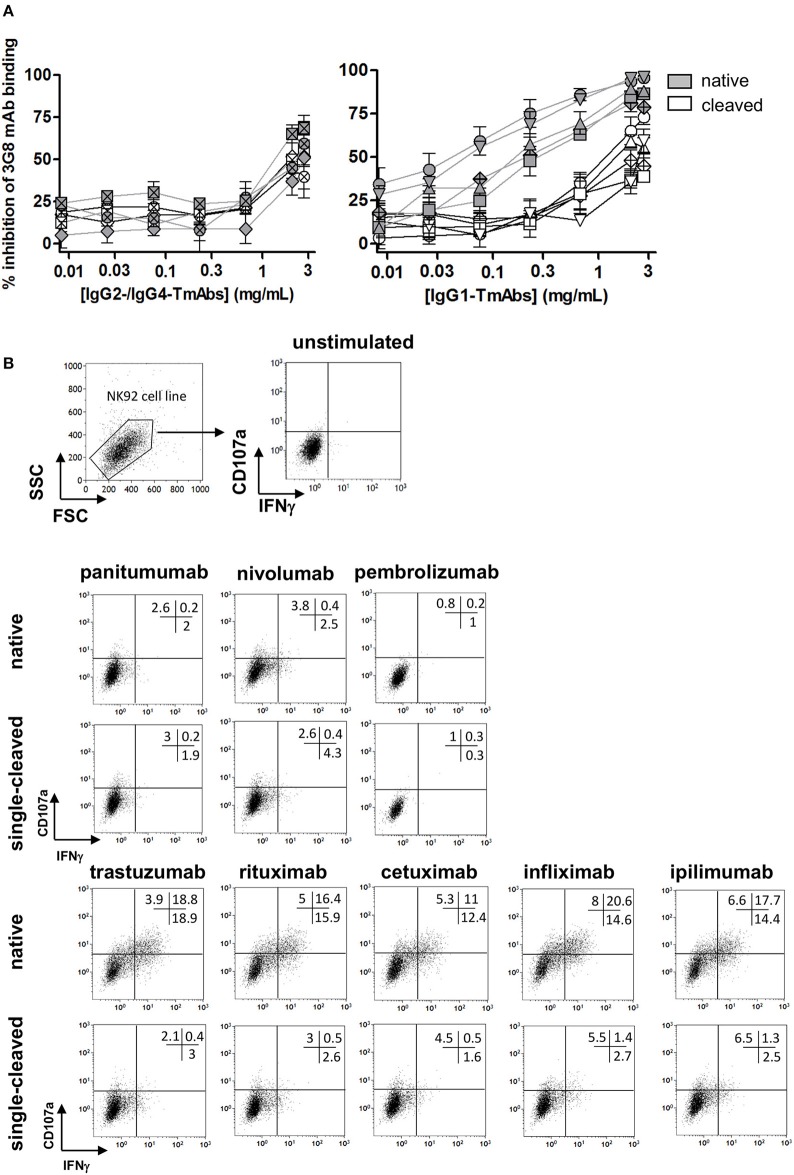
Binding of sc-TmAbs to FcγRIIIA-transduced NK92 cells and FcγRIIIA-dependent functional responses. **(A)** Binding of cleaved or intact IgG2/IgG4 TmAbs (left) or IgG1 TmAbs (right) to FcγRIIIA-transduced NK92 cells. Percentage inhibition of 3G8 binding was analyzed as described previously (18). The eight TmAbs were used: trastuzumab (circle/TTZ), cetuximab (triangle/CTX), infliximab (crossed diamond/IFX), ipilimumab (square/IPI), rituximab (inverted triangle/RTX), panitumumab (diamond/PAN), nivolumab (crossed circle/NIV), and pembrolizumab (crossed square/PEM). **(B)** Gating strategy and flow cytometry analysis of FcγRIIIA-transduced NK92 cells incubated in the absence or presence of intact or cleaved plate-bound IgG2 or IgG4 TmAbs (row 2 and 3) or IgG1 TmAbs (row 4 and 5). The percentages of degranulation and IFNγ-producing cells are shown. Results are representative of three different experiments.

We then compared the effect of single cleavage on binding of the TmAbs to the FcγRIIIA expressed on NK cells. We measured the inhibition of the FITC-conjugated anti-CD16 mAb (3G8) binding to FcγRIIIA/CD16^+^ NK92 cells by the intact TmAbs and sc-TmAbs. Intact IgG2 and IgG4 TmAbs poorly competed with binding of the 3G8 (Figure 4A, left), which shows their extremely weak binding to membrane FcγRIIIA. Accordingly, we did not detect an effect of the cleavage on FcγRIIIA binding, because the curves obtained with sc-TmAbs and their respective intact forms were similar. The inhibition curves revealed that the five intact IgG1 TmAbs bound efficiently to FcγRIIIA (Figure 4A, right). The curves were similar, starting at 0.03 mg/mL and reached maximal inhibition (≈80%) at 3 mg/mL. The binding of the single-cleaved IgG1 TmAbs was substantially reduced because the inhibition was detected at 0.3 mg/mL and reached an upper limit (at 3 mg/mL) of 35–60% depending on the TmAbs (Figure 4A, right).

We then compared the ability of sc-TmAbs to trigger NK functional responses upon FcγRIIIA engagement. Given the lack of a cell expressing all antigens (at similar level) recognized by the eight TmAbs, we could not compare the functional responses in a target cell-based assay (such as ADCC). Therefore, we used a target cell-independent assay with FcγRIIIA-expressing NK92 cells incubated in plates sensitized overnight with a saturating concentration of intact or sc-TmAbs and analyzed by flow cytometry (Figure 4B, row 1). In accordance with their low affinity for FcγRIIIA, degranulation and IFNγ production were low after incubation with intact or single-cleaved forms of IgG2 and IgG4 TmAbs (Figure 4B, row 2 and 3). By contrast, incubation with intact IgG1 TmAbs resulted in substantial responses. The percentages of responding cells were decreased (4–5-fold lower) after incubation with the corresponding single-cleaved forms (Figure 4B, row 4 and 5).

### Effect of a Single Cleavage of TmAbs on C1q Binding

**Figure 5:**
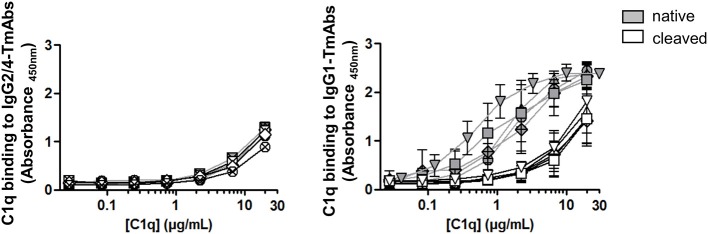
C1q binding on sc-TmAbs. ELISA of C1q binding for the eight TmAbs: trastuzumab (circle), cetuximab (triangle), infliximab (crossed diamond), ipilimumab (square), rituximab (inverted triangle), panitumumab (diamond), nivolumab (crossed circle), and pembrolizumab (crossed square). C1q concentrations from 0.01 to 30 μg/mL were incubated in plates sensitized with intact or single-cleaved TmAbs at saturating concentration. Horseradish peroxidase-conjugated anti-C1q was added to each well, then TMB as a substrate after 1 h, and absorbance was read at 450 nm. Data are mean (SD) for binding on IgG2/IgG4 TmAbs (left) or IgG1 TmAbs (right) (*n* = 3).

We then compared the effect of single cleavage of the eight TmAbs on the binding of C1q. We used an ELISA method with increasing concentrations of C1q added to plates sensitized with intact or single-cleaved TmAbs. The binding of C1q to intact or single-cleaved forms of IgG2 and IgG4 TmAbs was, as expected, extremely weak, detected at C1q >2 mg/mL (Figure 5, left). Conversely, we detected C1q binding to the intact IgG1 TmAbs at C1q ≥0.2 mg/mL and peaking at ≈10 mg/mL (Figure 5, right). The binding of C1q to rituximab was slightly higher of its binding to the other IgG1 TmAbs. The binding of C1q to the single-cleaved IgG1 TmAbs was similar and was decreased by about 10-fold as compared with the binding to their intact counterpart. In this case, the binding was detected at C1q ≥3 mg/mL without reaching a maximum even at C1q ≥20 mg/mL.

## Discussion

To the best of our knowledge, this study is the first to compare kinetically the cleavage of the lower hinge region of a panel of TmAbs. Our results clearly demonstrate that TmAbs with the IgG1 and IgG4 format are both MMP12- and IdeS-sensitive, whereas a TmAb with an IgG2 format was more resistant. Comparing ipilimumab to the other IgG1 TmAbs or pembrolizumab to nivolumab showed that their cleavage kinetics on incubation with a given protease differed substantially and different ranking with IdeS and MMP12 incubation. Moreover, our results indicate that the specific cleavage kinetics of ipilimumab were unrelated to its allotype and did not extend to its Fc portion. We have also shown that the affinity for C1q and FcγRIIIA of all IgG1 TmAbs decreased greatly after a single cleavage, reaching the level observed with IgG2 or IgG4 TmAbs. Accordingly, IgG1 sc-TmAbs lost their FcγRIIIA-dependent functional responses. Our results show that the cleavage kinetics by proteases present in pathological microenvironments should be considered in the design and development of TmAbs.

Several previous studies have shown that monoclonal IgG2 were almost completely resistant to cleavage by MMPs (11, 14). By contrast, their first HC was cleaved by IdeS, whereas the cleavage of their second HC was incomplete, as revealed by the simultaneous presence of sc-IgG2 and F(ab')_2_ after 24 h of incubation (11). Our results confirmed that the IgG2 mAb panitumumab was MMP12-resistant and that the cleavage of its second HC by IdeS was reduced as compared with that of the other TmAbs. We could not compare different IgG2 TmAbs in our study and therefore cannot state that this is a general feature of these molecules. Replacing amino acids from an IgG1 hinge region with those of an IgG2 led to protease resistance (14), so the relative resistance of IgG2 is probably mainly, if not exclusively, dependent on the specific sequence of its lower hinge region.

All the other TmAbs we tested were cleaved, although at different levels by IdeS and MMP12. The cleavage kinetics of the two proteases differed. Indeed, as previously described, IdeS cleaved the first heavy HC extremely rapidly. F(ab')_2_ was observed afterward, which indicates that the cleavage of the second HC occurred mainly on sc-TmAbs (21). Furthermore, this second cleavage was slow and incomplete even after 24 h. By contrast, MMP12 cleaved the first HC more slowly, and sc-TmAbs and F(ab')_2_ were at a similar level at 4–6 h, which indicates that the cleavage of the two HCs occurred simultaneously. In addition, sc-TmAbs were no longer detected after 24 h (except for ipilimumab), so the cleavage of the second HC was faster and more complete than that observed with IdeS. In addition, we observed two additional bands at 24 h when IgG1 TmAbs were incubated with MMP12. These bands were detected after sc-TmAbs disappeared. They probably correspond to a third cleavage occurring on the F(ab')_2_ in the upper hinge region (data not shown). Therefore, MMP12 can cleave the upper hinge region in the absence of the Fc region (Figure 1), whereas it was unable to cleave the lower hinge region in the absence of Fab (as shown in Figure 3 by the very low cleavage of the Fc portion of IgG1 TmAbs). Conversely, it has been proposed that IdeS interacts with the CH2 domain, before cleaving the hinge (8). This is in accordance with our finding that IdeS cleaved the Fc portion and the intact form of IgG1 TmAbs (excluding ipilimumab) with very similar kinetic (Figures 1D, 3D). Hence, our results indicate that IdeS and MMP12 have a different site of interactions with IgG leading to different kinetics.

Using both proteases, we compared the cleavage of five IgG1 and two IgG4 TmAbs. Ryan et al. suggested that IgG1 and IgG4 mAbs were similarly susceptible to MMP3 and IdeS cleavage (6), and the latter has been shown to cleave IgG4 down to F(ab')_2_ (22). Our results showed that the two IgG4 TmAbs tested were cleaved by MMP12 and IdeS. However, pembrolizumab and nivolumab were poorly and very strongly cleaved by IdeS, respectively, whereas both were highly cleaved by MMP12 (and more rapidly than all the IgG1 TmAbs tested). Thus, the sensitivity/resistance of TmAbs with the IgG4 format may differ substantially depending on the TmAb and the protease. Both TmAbs have the S228P mutation (6), which rules out that this structural characteristic may explain their differences in cleavage. The crystallographic resolution of pembrolizumab showed that one CH2 domain was rotated ~120° as compared with other known mAbs and the authors suggested that this new conformation may not be a crystallization artifact (23). Thus, assuming that the CH2 domain rotation is actually indicative of the structure of pembrolizumab in free solution and considering that IdeS must bind to the CH2–CH3 interface to cleave the hinge region (8), it may be hypothesized that the rotated CH2 of pembrolizumab could account for its reduced IdeS-mediated cleavage. The variability in cleavage kinetics observed with IgG4 TmAbs was confirmed when comparing TmAbs with the IgG1 format. Indeed, ipilimumab was strongly MMP12-resistant and IdeS-sensitive, whereas the other four IgG1 TmAbs were cleaved with similar and intermediate kinetics by each protease. Several previous studies have reported that monoclonal IgG1 molecules are cleaved efficiently by MMP3, MMP7, MMP12, and IdeS (6, 9, 10). Our results demonstrate that this is not a general feature. Even though the amino acid composition of the lower hinge region is clearly involved in the resistance of IgG2, the sequence is the same in a given subclass (i.e., the five IgG1 TmAbs and two IgG4 TmAbs tested). Therefore, the differences in the cleavage kinetics with the IgG1 and IgG4 TmAbs are due to other characteristics.

We hypothesized that the variability observed for ipilimumab among IgG1 TmAbs was due to its G1m3;1 allotype. Indeed, we have recently shown that the IgG1 allotype affected the affinity of an mAb for the FcRn, showing that a structural variation located outside an interaction site may nevertheless affect this interaction (17). However, we observed that the four allotypes of an mAb constructed on the basis of rituximab were cleaved similarly, which suggests that this polymorphism does not account for the feature of ipilimumab. It may be assumed that the glycans present in position N297 are involved in the cleavage modulation because glycosylation can affect the cleavage of the upper hinge region of IgG by papain (24). However, the binding of TmAbs to FcγRIIIA is influenced by the glycan structure (25), whereas we found similar binding of all tested IgG1 TmAbs including ipilimumab. Moreover, our results showed that the cleavage of ipilimumab Fc fragment by IdeS was superimposable to that of the 3 other IgG1 TmAbs Fc fragment. We conclude that the variability observed for intact ipilimumab is not related to natural structural differences located within its Fc portion (including glycosylation). We therefore propose that the Fab region could affect the cleavage kinetics of a given TmAb.

Among variations of the Fab related to bioengineering, the type of humanization could be important. In line with this assumption, ipilimumab was the sole fully human IgG1 tested in our study, whereas the other IgG1 TmAbs were chimeric or humanized. The fact that nivolumab and ipilimumab, which are both fully human TmAbs, were cleaved with opposite kinetics by MMP12 indicates that this type of engineering is not associated with a given cleavage sensitivity/resistance profile. Overall, our results confirm that the cleavage sensitivity/resistance by proteases might mainly depend on the subclass when comparing IgG2 vs. IgG1 or IgG4 TmAbs. They demonstrate for the first time a variability in this sensitivity/resistance balance among TmAbs of IgG1 and IgG4 subclasses, which likely results from TmAb characteristics probably related to and/or located in the Fab region and may be at least partially specific to a given TmAb.

The functional consequences of the proteolytic cleavage have been broadly studied with mAbs including TmAbs such as trastuzumab or pertuzumab, in *in vitro* and *in vivo* models (12, 13). We explored the ability of the eight TmAbs to bind to the membrane form of FcγRIIIA. A single cleavage decreased the binding, whatever the tested IgG1 TmAbs, in accordance with the results reported with trastuzumab by using ELISA with recombinant FcγR (12). Consequently, IgG1 sc-TmAbs were not able to induce FcγRIIIA-dependent IFNγ production and degranulation by NK92 cells on stimulation with plate-bound TmAbs, as compared with their intact forms. Due to the lack of a cell expressing all antigens recognized by the TmAbs tested, we could not compare the functional responses in a target cell-based assay. We however observed that IgG2 and IgG4 TmAbs, which are inefficient to mediate ADCC or CDC due to their reduced affinity for FcγRIIIA and C1q (confirmed in our study), were also inefficient to induce degranulation or cytokine production in our target cell-independent assay, conversely to intact IgG1 TmAbs. NK-cell degranulation assessed by CD107a labeling has been widely used as a surrogate marker for cytotoxic activity (26–28) including rituximab- trastuzumab- or cetuximab-mediated ADCC directed against target cell lines expressing CD20, Her-2 or EGRFR, respectively. The fact that IgG1 sc-TmAbs were inefficient as compared to intact forms in our assay, strongly suggest that the lack of activation would extend to functional responses (such as depletion) observed in target cell dependent assays. FcγRIIIA expression is not restricted to human NK cells. Several FcγRIIA-expressing myeloid cells, including a subset of monocytes, macrophages, and neutrophils (at low levels) (29, 30) co-express FcγRIIIA. Both receptors are involved in myeloid cell-mediated functions such as ADCC, antibody-dependent cellular phagocytosis (ADCP) and trogocytosis (31–33). Several lines of evidence indicate that myeloid cells contribute substantially to the mechanism of action of cytolytic tmAbs [reviewed in (34)]. Indeed, targeting the myeloid-specific immune checkpoint CD47/SIRP-a pathway, triggers ADCP mediated by anti-CD20 and anti-Her2 mAbs *in vitro* and results in increased antitumor activity of TmAbs in diverse mouse models. The H131R polymorphism of *FCGR2A* gene encoding FcγRIIA, (whose expression is restricted to myeloid cells), is associated with patient responses to rituximab (lymphoma), cetuximab (colon cancer), and trastuzumab (breast cancer). Finally, a high tumor-associated macrophage infiltration is associated with favorable outcome in follicular lymphoma and breast cancer patients treated with rituximab and trastuzumab, respectively. Our results confirmed that the cleavage of a single H dramatically reduced the binding of IgG1 TmAbs to FcγRIIIA. It has been reported that it decreased similarly the binding to FcγRIIA and partially abrogated FcγRIIA-dependent functions induced by TTZ (12). Therefore, it may be assumed that the reduced FcγRIIIA-dependent functions mediated by NK cells reported herein with IgG1 sc-TmAbs, probably extend to FcγRIIIA- and FcγRIIA-dependent functions mediated by myeloid cells. This assumption is sustained by the fact that peripheral blood mononuclear cells (instead of NK cells) were used as effector cells, in the previous studies showing the reduced lysis mediated by sc-TmAbs compared with intact TmAbs (12, 13). Further studies are warrantied to evaluate specifically the impact of the cleavage on FcγRIIA-mediated function such as ADCP. In addition, we observed impaired binding of C1q to IgG1 sc-TmAbs, which further suggests that the lower hinge cleavage may be an escape mechanism in cytolytic mAbs therapy.

The impact of the cleavage in TmAbs-based therapies is presently unknown. The cleavage of trastuzumab or pertuzumab has been associated with a weakened therapeutic effect in mouse xenograft tumor model (12, 13). In humans, increased tumor sc-IgGs were found to be associated with poor breast cancer patient outcomes (35) and high levels of MMP3-/MMP12-cleaved IgG were detected in sera from inflammatory bowel diseases patients, who did not respond to anti-TNFα therapy (10). These studies suggest that proteolytic degradation may contribute to compromised humoral immunity and/or non-responsiveness of patients to TmAbs. It is however of note that sc-IgG were detected in these studies, whereas, to the best of our knowledge, the presence of cleaved TmAbs in patients has not been reported so far. Our results underline the need to develop new assays to measure the levels of cleaved TmAbs *in vivo*, in order to evaluate/monitor the importance of the proteolytic cleavage in their efficiency. The loss of interaction with immune effectors by IgG1 TmAbs after a single cleavage showed that this subclass loses its advantage over IgG2 or IgG4 TmAbs in terms of cytolytic activity. Ipilimumab, whose antitumor effect depends on the Fc region (although it is an antagonist) (36) was almost uncleaved by a relevant protease such as MMP12. The IgG2 or IgG4 format is favored in the case of neutralizing/antagonist TmAbs, which do not require Fc-mediated functions. However, a second cleavage producing F(ab')_2_ would negatively affect the pharmacokinetics of these antibodies, which would be eliminated more quickly because of an absence of binding to FcRn. With these potential consequences, our results show the need to study each TmAb in the relevant protease microenvironment to assess its susceptibility/resistance balance. Even though non-cleavable structures are being developed by mutating the hinge region (14), we show that a natural format can resist the cleavage without structure modification. A better understanding of this phenomenon is required to limit the escape mechanism in TmAbs-treated patients.

## Data Availability Statement

All datasets generated for this study are included in the article/supplementary material.

## Author Contributions

QD, LL, and BB designed and performed the experiments. QD, LL, BB, and GT analyzed the results. QD, LL, and GT wrote the manuscript. LL and GT supervised the study design and GT supervised the study conception. All authors critically revised the work, provided substantial input, and gave final approval to the version to be published.

### Conflict of Interest

The authors declare that the research was conducted in the absence of any commercial or financial relationships that could be construed as a potential conflict of interest.
